# Influence of seasonality and gestation on habitat selection by northern Mexican gartersnakes (*Thamnophis eques megalops*)

**DOI:** 10.1371/journal.pone.0191829

**Published:** 2018-01-30

**Authors:** Tiffany A. Sprague, Heather L. Bateman

**Affiliations:** College of Integrative Sciences and Arts, Arizona State University, Mesa, Arizona, United States of America; University of Regina, CANADA

## Abstract

Species conservation requires a thorough understanding of habitat requirements. The northern Mexican gartersnake (*Thamnophis eques megalops*) was listed as threatened under the U.S. Endangered Species Act in 2014. Natural resource managers are interested in understanding the ecology of this subspecies to guide management decisions and to determine what features are necessary for habitat creation and restoration. Our objective was to identify habitat selection of northern Mexican gartersnakes in a highly managed, constructed wetland hatchery. We deployed transmitters on 42 individual gartersnakes and documented use of habitat types and selection of specific habitat features. Habitat selection was similar between males and females and varied seasonally. During the active season (March–October), gartersnakes primarily selected wetland edge habitat with abundant cover. Gestating females selected similar locations but with less dense cover. During the inactive season (November–February), gartersnakes selected upland habitats, including rocky slopes with abundant vegetation. These results of this study can help inform management of the subspecies, particularly in human-influenced habitats. Conservation of this subspecies should incorporate a landscape-level approach that includes abundant wetland edge habitat with a mosaic of dense cover for protection and sparsely vegetated areas for basking connected to terrestrial uplands for overwintering.

## Introduction

One of the most important elements of species management and conservation is knowledge of habitat requirements [1,2]. Understanding habitat selection at multiple spatial scales is vital for effective species management [3,4]. Habitat selection in ectothermic animals can be driven by intrinsic factors, such as body size and reproductive condition [5–7], and extrinsic factors, such as distribution of resources, temperature, predators, and prey [8–10]. Selection of habitat features is not static because animals can alter selection based on daily or seasonal variation [11,12]. In snake species, behaviors such as hibernating, breeding, and foraging can strongly influence habitat selection [1,13,14]. Habitat modification and loss can restrict the ability of animal species to move about the landscape to preferentially select required resources [15,16]. Many species of snakes have experienced dramatic population declines because of habitat loss and degradation [17,18], including species that rely on wetland areas [13,19]. Rivers and wetlands around the world have been disrupted and fragmented, affecting the rich biodiversity that depends on them [20–22]. Many species of aquatic and semi-aquatic snakes have declined due to drought and human-caused impacts to their habitat [18,23,24].

In the semiarid southwestern United States, riparian areas are imperiled habitat [25,26] and occupy less than 3% of the total land area [27]. However, riparian areas provide a mosaic of productive habitats and support many species [19,28]. Numerous aquatic and semi-aquatic animal species have declined due to damming and diversion of surface water and pumping of groundwater [29,30]. One hallmark subspecies that has experienced such declines is the northern Mexican gartersnake (*Thamnophis eques megalops*; S1 Fig). Substantial portions of the historical range of the northern Mexican gartersnake have been dewatered, resulting in local extirpations [31,32]. Many sites where this gartersnake persists have been reduced in size or have become isolated [31–33]. Historically, the northern Mexican gartersnake ranged throughout much of central and southern Arizona, into southwestern New Mexico and Mexico, and may have occurred in California and Nevada along the Colorado River [32]. The subspecies now occurs at low densities and may be extirpated from as much as 90% of its historical range in Arizona and New Mexico [31–33]. The northern Mexican gartersnake is state-listed and federally listed as threatened [32,34,35].

The few studies on this subspecies have described home ranges and general habitat associations (second and third-order habitat selection based on [36] and [37]). Third-order habitat selection of gartersnakes has been described as protected backwaters, pools, cienegas, stock tanks, and stream edges rich with emergent vegetation [33,38,39]. Gartersnakes have also been documented in human-modified areas, such as fish hatcheries. Boyarski et al. [40] documented gartersnakes spending active seasons on hatchery pond edges and in cattail-dominated areas and overwintering in upland habitat composed of rocky, shady slopes. However, within these larger-scale descriptions, little is known about selection of fine-scale structural features and sites (e.g., ground cover, vegetation, and substrate of fourth-order selection [36]). Many species of snakes select areas based on fourth-order habitat parameters, which are often more important than third-order habitat features for thermoregulation, predator avoidance, and foraging [7,10].

Similarly, little is known about seasonal variation in habitat selection of northern Mexican gartersnakes during the active, gestation, and inactive seasons. Many animal studies focus on habitat use during only one season and may exclude animals based on reproductive condition or maturity. Such limitations provide an incomplete picture of the full habitat needs of a species. Semi-aquatic species, such as the northern Mexican gartersnake, rely on both aquatic and terrestrial habitats [39–41]. Understanding the spatial and temporal uses of habitat by gartersnakes is critical for effective habitat conservation [42,43] by informing the size and type of areas to be conserved. Knowledge of these habitat requirements can guide the timing and location of management activities to minimize adverse effects or disturbance to the subspecies [44,45].

The objective of our study was to identify habitat parameters selected by northern Mexican gartersnakes. We examined habitat features selected by gartersnakes during the active, gestation, and inactive seasons and compared habitat differences due to sex. We expected to document seasonal differences in habitat due to a separation in overwintering and foraging requirements and also expected to see some differences between genders due to the larger body size and reproductive needs of females. Because northern Mexican gartersnakes are viviparous [38], thermoregulation may be more important during gestation than foraging. Therefore, we compared habitat requirements during the gestation period to active and inactive periods. Results of this study will help resource managers understand specific habitat features used by this subspecies during different seasons and across life-history stages. By understanding the spatial and temporal ecology of this subspecies, managers can use this information to maintain or construct features to provide suitable habitat.

## Methods

### Study site

**Fig 1:**
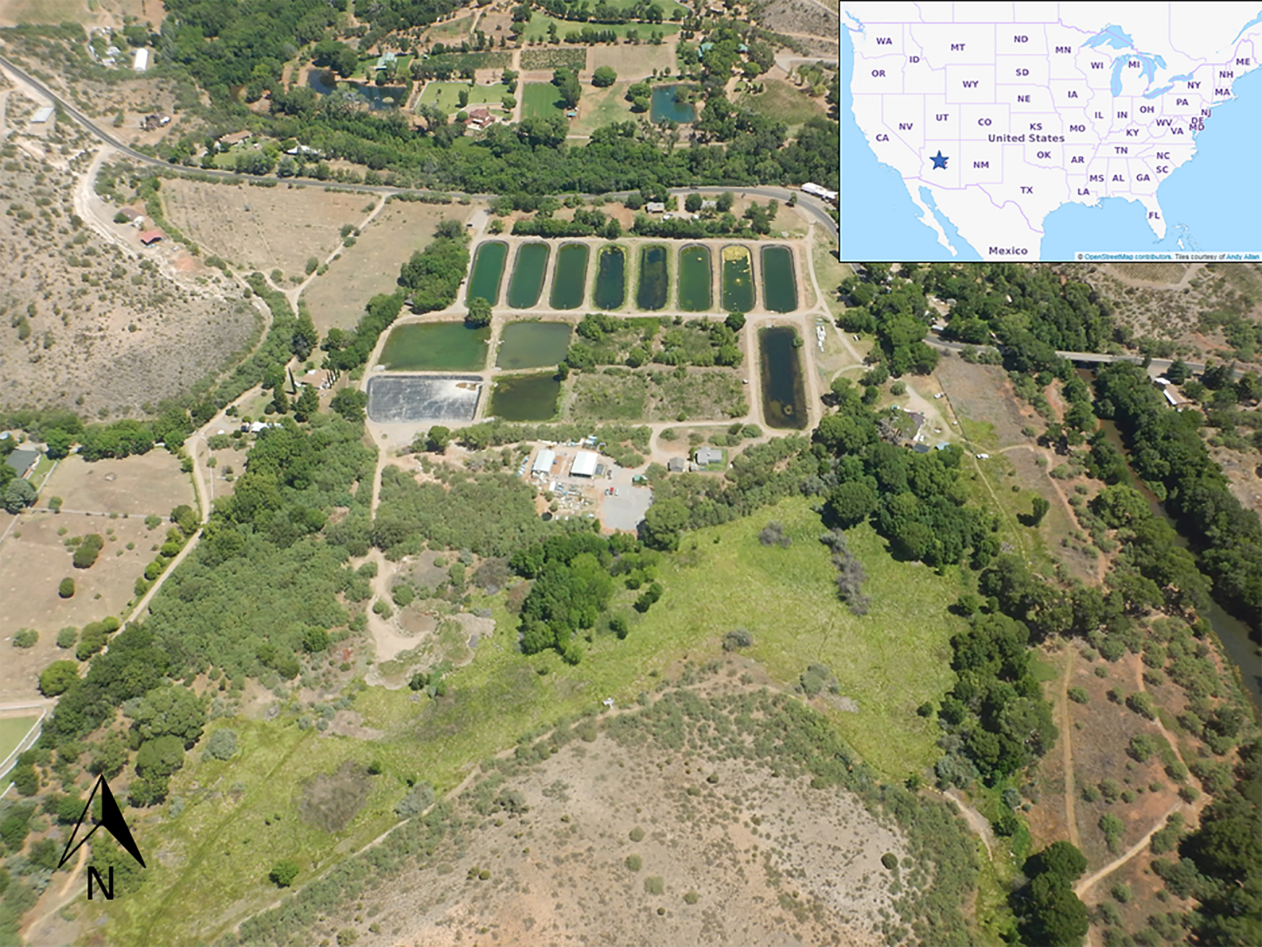
Study site: Bubbling ponds hatchery in Yavapai County, Arizona, looking north. Active fish-rearing ponds are the nine long oval ponds to the north and east. Fallow ponds are the four vegetated blocks in the south middle. The four ponds to the southwest were drained during much of the study (June 2015 –February 2016). The pond in the far southwest was lined with black polypropylene liner and remained empty. To the south of the managed ponds are a rocky ridge covered by trees and a wet meadow. Oak Creek borders the site on the east. Inset shows general location (star) of the study site in North America.

Our study area was a 21.9-ha state-managed fish hatchery described as sustaining one of only five viable populations of northern Mexican gartersnakes in the United States [32]. Bubbling Ponds State Fish Hatchery (hereafter, the hatchery), located in Yavapai County, Arizona, (UTM NAD83 0418091E 3847618N) raises warm water native fishes and introduced sportfish. The Arizona Game and Fish Commission purchased the property in 1954 and has operated it as a fish hatchery since 1955. Elevation ranges from 1052–1180 m. The hatchery includes lined and unlined fish-rearing ponds, fallow ponds no longer used for fish production, meadows dominated by sedges and grasses, mesquite (*Prosopis velutina*) and riparian woodlands, and dense thickets of non-native blackberry (*Rubus* sp.; Fig 1). Other vegetation at the hatchery includes a mix of native and non-native species. The hatchery is bordered by Oak Creek and hills of semidesert grassland and mixed evergreen–deciduous shrubland [46,47].

### Field methods

We captured gartersnakes using a combination of Gee^™^ minnow traps [31], coverboards [48], and visual encounters. We deployed 50–100 traps per day from May–October 2015 and April–October 2016 for 4,966 trap days. Traps were checked and emptied twice a day. Some nontarget species (anurans, salamanders, fish, and insects) were left in traps to serve as bait. We marked, measured, and sexed each captured northern Mexican gartersnake. We marked all gartersnakes using cautery branding [49] and microchipped gartersnakes >25g with PIT tags [50,51].

We employed radio telemetry to monitor animal movement and habitat selection [10,52,53]. We used a combination of internal and external transmitters on a total of 42 individual gartersnakes. A veterinarian surgically implanted temperature-sensing transmitters (SB-2T [5.2g] or BD-2T [1.9g], Holohil Systems Ltd., Ontario, Canada) in 22 individual gartersnakes. We followed recommended surgery and post-operative care *sensu* [39]. We fitted external transmitters (BD-2 or temperature-sensing BD-2T units; 1.8g, Holohil Systems Ltd., Ontario, Canada) on an additional 20 gartersnakes using tape [54]. Eight gartersnakes received more than one type of transmitter (internal/external) over the course of the study. Transmitters were no more than 5% of the gartersnake’s mass at the time of deployment. We released gartersnakes at their capture locations, unless they had been captured inside a construction area, in which case they were released into adjacent fallow ponds. We brought transmittered gartersnakes that exhibited signs of illness to a veterinarian for care, and all functioning transmitters were removed from gartersnakes by the end of the project. Our research was covered under the following permits: US Fish and Wildlife Service (AGFD Section 6 and TE43322B-0), and Arizona Game and Fish Department (SP733846 and SP744166). This study was carried out in accordance with protocol approved by Institutional Animal Care and Use Committee from Northern Arizona University (Permit NAU 14–010) during 2015 and from Arizona State University (Permit ASU 15-1417R) during 2016. All surgery was performed under anesthesia by experienced and licensed veterinarians, and all efforts were made to minimize suffering.

We located transmittered gartersnakes at least once per week from May 2015 through August 2016. To ensure individuals were located during different diel periods, we assigned gartersnakes to tracking cohorts, which we tracked weekly at different times on a rotating basis (i.e., early day [0700–1100], midday [1100–1500], and late day [1500–1900]). Trained observers located transmittered gartersnakes to within 30 cm, although snakes occasionally flushed before their location could be pinpointed, and underground signals may have been distorted by roots or rocks. We field tested position accuracy by locating 100% (n = 31) of shed external transmitters. Because gartersnakes were frequently underground or relied on procrypsis when aboveground, we were able to pinpoint locations without flushing snakes more than 97% of the time. Each location, hereafter referred to as the snake point, was recorded using a global positioning system (GPS) unit (Garmin Ltd., Schaffhausen, Switzerland) and was marked with flagging tape to identify the exact location of the gartersnake. We also recorded GPS location accuracy, whether or not the gartersnake was visible, snake behavior (if observable), and transmitter pulse rate (used to calculate body temperature).

We divided sampling into three seasons: active (March–October), gestation (April–May for females only), and inactive (November–February). Inactive season was determined for each individual based on amount of movement and when each snake entered its overwintering habitat. Gestation period was based on females known to be pregnant. Because we used a hands-off approach to minimize influence on behaviors and habitat selection, we could not confirm reproductive status for all females in 2016. However, all females captured during May 2015 (n = 7) and more than half (n = 6) of transmittered females in April–May 2016 were confirmed to be pregnant. We determined the start of the gestation season based on enlarged ovaries observed by a veterinarian during transmitter implant surgery in early April 2016 and ultrasounds of embryos in May 2015. The end of gestation season was based on observation of neonates during the first week of June in 2015 and 2016. Therefore, we determined the gestation season to be April–May and included all females in the gestation habitat assessment.

### Habitat assessment

**Fig 2:**
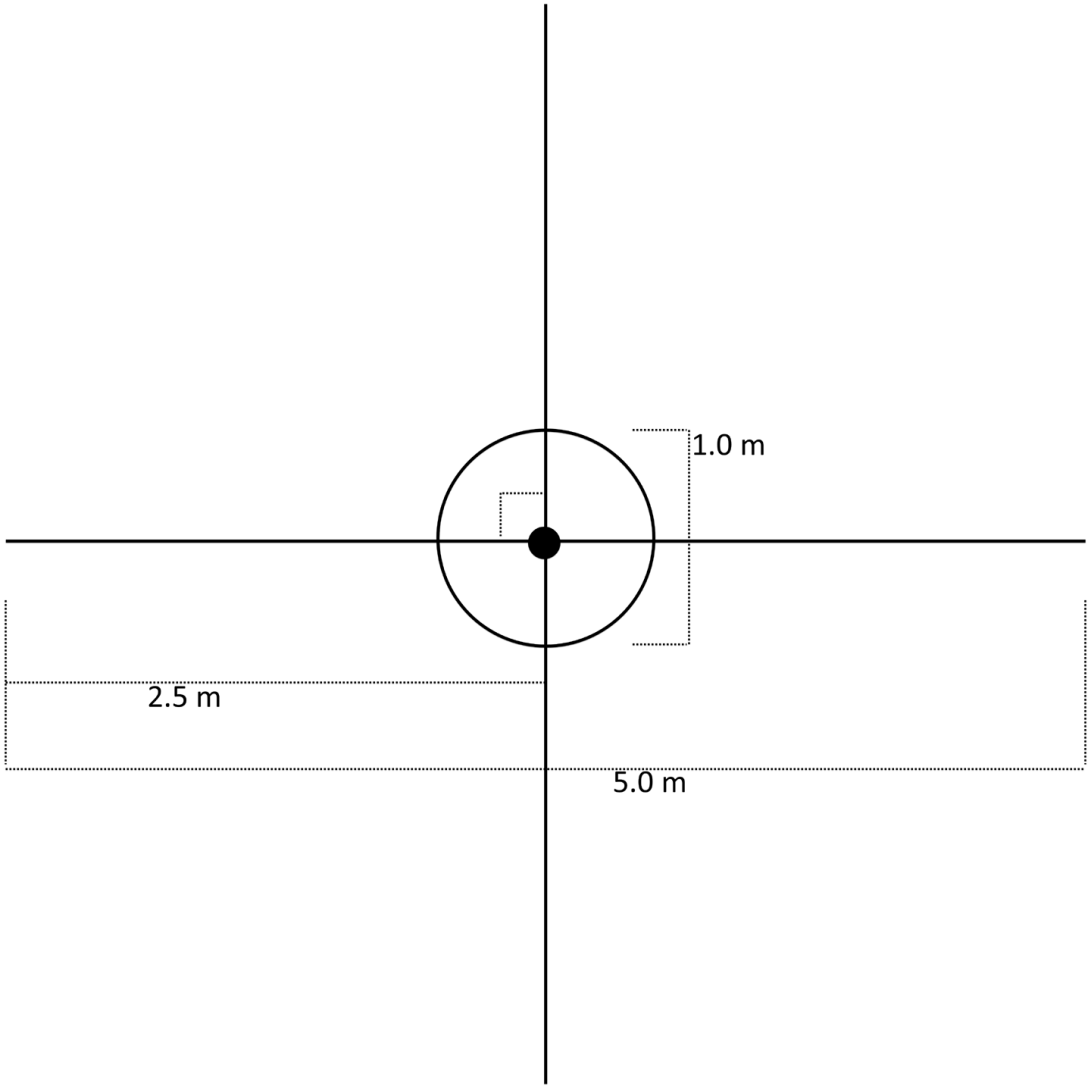
Diagram of plot and transect design used to measure habitat variables. One 1-m-diameter plot and four randomly-oriented perpendicular 2.5-m transects placed with the snake/random location as the centerpoint.

**Fig 3:**
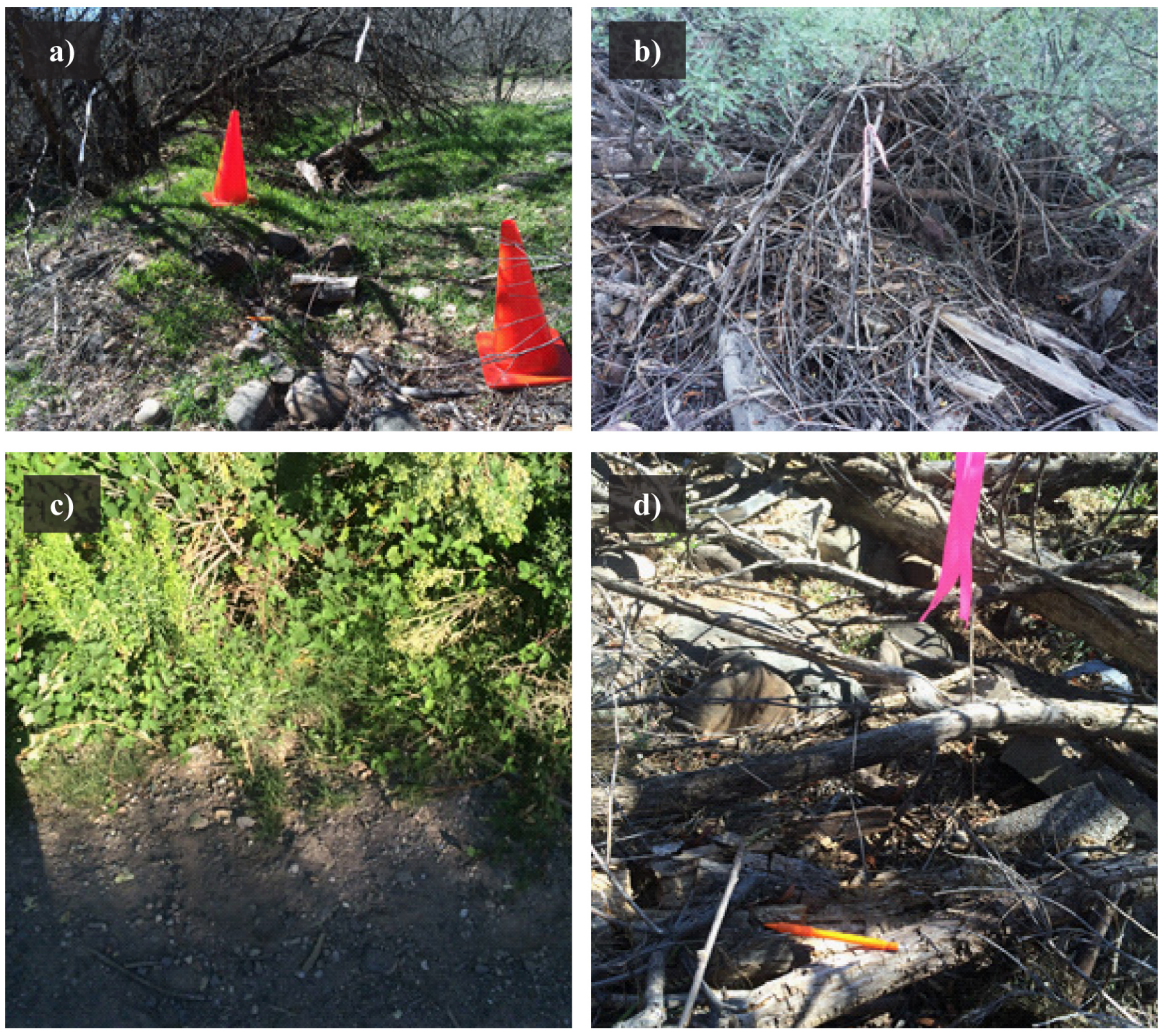
Examples of cover types. a) Canopy cover from trees, low-height cover from living vegetation (grass), and ground cover from bare, rock, litter, woody debris, and small-diameter vegetation; b) low-height cover from living and dead vegetation, litter, and woody debris and ground cover from litter and woody debris; c) low-height cover from living vegetation (forb and grass) and ground cover from bare, rock, and small-diameter vegetation; d) low-height cover from vegetation, woody debris, and litter and ground cover from rock, litter, and woody debris.

We measured habitat where we found gartersnakes through tracking and visual observations. Fourth-order habitat measurements included vegetative, environmental, and hydrologic characteristics (Table 1) recorded at each snake point, in a 1-m-diameter plot, and along four 2.5-m transects *sensu* [10,39,55,56] (Fig 2). At each snake point, we measured aspect and slope, water depth, distance to water, and canopy cover (>1m in height). Within a 1-m-diameter plot centered on the snake point, we recorded number of plant stems (≥1 cm diameter) rooted in the plot and percentages of ground cover type, submerged vegetation, and surface shade. In plots, we also recorded percentage of low-height cover (≤1 m), which included vegetation (living or dead), woody debris, deep loose litter, or human-made structures that a snake could use for potential cover. We defined ground cover as anything a snake could be on top of when aboveground (Fig 3). We used ocular estimates of cover classes [57] in the following percentages: 0, <1, 1–5, 5–25, 25–50, 50–75, 75–95, >95. On four intersecting 2.5-m transects, we quantified vegetation type (grass, forb, cattail, sedge/rush, shrub, tree, or none) at every 0.5m mark. We measured habitat only at unique snake locations, which excluded points <3 m from a previous location for that snake (to avoid overlap in measurements) that had been measured in <4 weeks [10,55].

**Table 1:** Habitat characteristics.

Method/Variable	Description
*Point*	*Recorded at snake/random location*
Surface/water temperature	Temperature (°C) measured at ground or water surface
Air temperature	Temperature (°C) measured 1m above the ground
Relative humidity	Relative humidity (%) measured 1m above the ground
Aspect	Compass bearing (°) of slope
Slope	Slope of the immediate area
Canopy cover	Percent cover provided by vegetation >1 m in height (measured with a densiometer in four directions and then averaged)
Water depth	Depth (cm) of water (if point was in water)
Distance to water	Distance (m) to water from point
*Plot*	*Recorded in a 1-m-diameter circular plot with snake/random location at center*. *Cover and shade percentages were ocular estimates in the following classes*: *0*, *<1*, *1–5*, *5–25*, *25–50*, *50–75*, *75–95*, *>95%*.
Shade	Percent of surface shaded
Low-height cover	Percent cover ≤1m in height (what a snake would be under) provided by vegetation (living or dead), debris, deep litter, human-made objects
Submerged vegetation	Percent of area with submerged vegetation
Ground cover	Proportion of ground cover (what a snake would be on top of) classified as bare ground, rock, litter, woody debris (diameter ≥1cm), small vegetation (<1cm diameter), large vegetation (≥1cm diameter), water
Vegetation density	Number of plant stems ≥1cm diameter rooted in the plot; if in water, only emergent vegetation was counted.
*Point-intercept*	*Occurrence of vegetation at 0*.*5-m intervals along four randomly-oriented perpendicular 2*.*5-m transects with the snake/random location at the center*
Vegetation type	Percentage of vegetation type (grass, forb, cattail, rush/sedge, shrub, tree, aquatic, none). Total vegetation cover could exceed 100%.

Definitions and description of methods used to quantify fourth-order habitat features at gartersnake locations and random locations.

To compare used and available habitat, we quantified fourth-order habitat variables at snake points and paired random points [10,58]. This matched-pairs design is more robust than unmatched studies for assessing habitat selection because each random location represents a true absence [59–61]. This technique also controls for variation in environmental conditions and enables more accurate modeling of habitat selection by ensuring that each random location is available to that individual at that time [62,63]. We randomized the distance (between 5 and 155 m) and bearing of the paired point from each snake point using a random number generator. If a random point occurred on private land or in an area not accessible to snakes, a new location was determined.

### Activity range size

We estimated 95% minimum convex polygon (MCP) activity range sizes (second-order selection [36]) to better understand the amount of movement within each season (ArcGIS version 10.3, Esri, with ArcMET 10.3.1 v1 software extension). Although widely used, MCP provides a rough estimate of second-order selection and might include large areas not used by the animal [64,65]. Therefore, MCPs might not represent true activity ranges but are useful to understand total range and relative movements [66]. Because number of locations for each individual can influence activity range size, we only included snakes for which activity range size plotted against number of locations reached an asymptote [10]; we used all locations available for these snakes to estimate activity range.

### Statistical analyses

We tested data for normality and equal variance using R (version 3.1.2, The R Foundation for Statistical Computing) package “car.” We calculated mean and standard error for each habitat variable using R package “plyr” and Oriana 4 (Kovach Computing Services, Anglesey, Wales). For subsequent analyses, we converted aspect to a categorical variable (i.e., N, E, S, W) and used the median of each ocular estimate class. To visualize habitat, we used a Principal Component Analysis (PCA) to reduce variables into components using SPSS (version 23.0, IBM). Because PCA is most suitable for datasets with a low number of zeros, we only included variables for which <40% of values were equal to zero [67,68]. We scaled and centered data prior to running the PCA to account for varying units of measurement among variables. Components with an eigenvalue >1 were selected and plotted for visualization [69].

To assess habitat selection and to identify key environmental variables, we used matched pairs logistic regression [70] to compare each snake point to its random location (R package “survival”). We modeled habitat selection by gender and season. When generating habitat models, the first step was to select variables for inclusion. We tested variables for multicollinearity using pairwise comparisons (cutoff of r≥0.6) and variance inflation factors (cutoff of VIF≥10). We then generated univariate matched-pairs logistic regression models to assess the significance of each variable [70] using R package “survival.” Variables were considered significant at *p*<0.25 because some variables might not be significant on their own but are significant in conjunction with other parameters [70]. Variables exhibiting complete separation (i.e., all snake locations had zeros for that variable but some random points had non-zero values or vice versa) were omitted to avoid statistical problems in the logistic regression [71]. We fitted a multivariate model with all uncorrelated variables found to be significant during the univariate tests [70]. If two or more highly-correlated variables were significant in univariate tests, we ran separate multivariate models with one of those variables. Variables that were clearly non-significant (*p*>0.25) were removed from the multivariate models. In a stepwise fashion, we then added variables eliminated during preliminary univariate and multivariate tests back into the models, one at a time, to test for significance [70]. Any non-significant variables (*p*>0.25) were again removed. We repeated this process until we obtained final models in which all variables were significant [70].

We used a ranked multiple-model inference approach to obtain unbiased coefficients for variables determined by the final models [72]. All possible subsets were considered (R package “MuMIn”). The top model had a ΔAIC = 0, but we also considered all models with a ΔAIC<2. We calculated variable weights within each model and then summed across all models to obtain weighted coefficients for each variable. Because a one-unit increase in an explanatory variable is rarely practical for continuous data [70], we determined increases based on means and ranges for each variable to calculate odds ratios.

We used a one-tailed t-test [73] to compare mass of females and males. We compared gartersnake body temperature by season and month using two mixed-effects ANOVAs [73], one with season and sex as fixed effects and a second with month and sex as fixed effects; individual snake was included as a random effect in both. ANOVAs were conducted using R package “lme4.” These data met assumptions of normality and equal variance. Tests were considered significant at α≤0.05.

## Results

### Habitat assessment

Of the 42 transmittered gartersnakes, 25 were female and 17 were male (S1 Table). We located transmittered gartersnakes 781 times to assess habitat and removed locations that were not unique and one location from a female behaving abnormally due to illness. We quantified habitat variables at 510 gartersnake locations and 510 random locations, including 486 telemetry and 24 visual observations. Paired locations were grouped into three seasons: active (n = 348), gestation (n = 57), and inactive (n = 105). Gartersnakes were visible 24.1% of times located for fourth-order habitat assessment (23.0% during the active season, 56.1% during gestation, and 10.5% during the inactive season).

**Fig 4:**
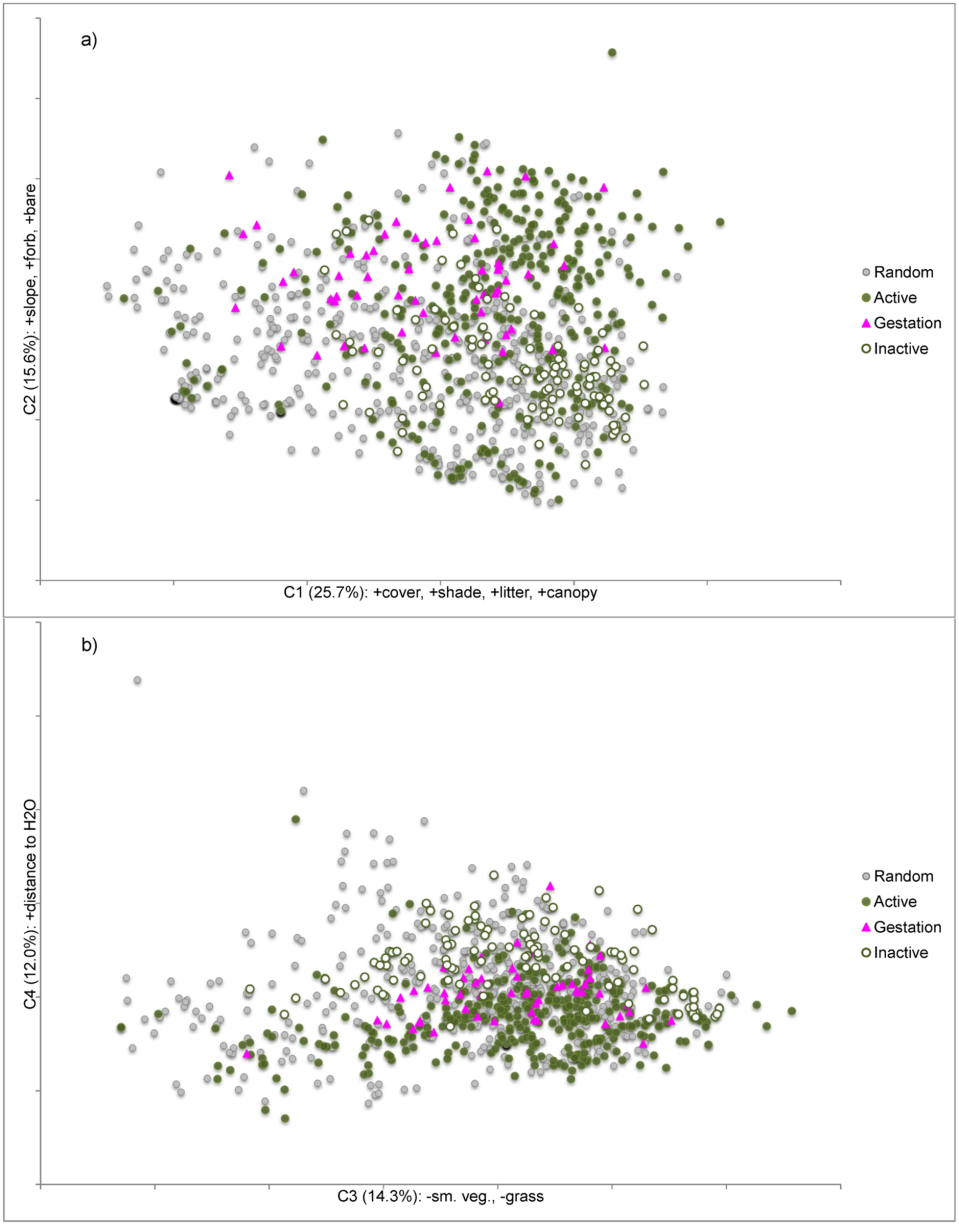
Biplots of four habitat components generated from PCA analyses. a) C1 (cover and litter) vs. C2 (slope, forb, and bare ground cover) and b) C3 (small-diameter vegetation abundance) and C4 (distance to water). Percentages in parentheses show the amount of variation in the data accounted for by that component.

Ten of 23 variables were included in the PCA: canopy cover, low-height cover, shade, bare ground cover, litter ground cover, small-vegetation ground cover, grass, forb, distance to water, and slope. These variables were reduced to four components that, when combined, explained 67.6% of variation in the data (Table 2). Component 1 described the most variation in habitat (25.7%) and represented elements of vegetative cover. Biplots of these components show high variability in habitat characteristics; however, gartersnakes displayed more narrow fourth-order habitat selection during the inactive season (Fig 4).

**Table 2:** Habitat described by four PCA components.

Variable	Component			
	1	2	3	4
Low-height cover (%)	**0.791**	-0.061	0.034	-0.395
Shade (%)	**0.777**	-0.102	0.107	-0.288
Litter ground cover (%)	**0.646**	-0.175	0.297	0.320
Canopy cover >1m high (%)	**0.515**	-0.447	0.302	0.241
Slope (°)	0.268	**0.674**	0.267	0.212
Forb (%)	0.353	**0.630**	0.340	-0.067
Bare ground cover (%)	-0.343	**0.470**	0.297	0.049
Ground cover, veg <1cm diam. (%)	0.339	0.192	**-0.782**	-0.276
Grass (%)	0.440	0.430	**-0.516**	0.323
Distance to water (m)	0.187	-0.132	-0.295	**0.752**
Variance explained (%)	25.7	15.6	14.3	12.0
Cumulative variance explained (%)	25.7	41.3	55.6	67.6

Principal Component Analysis (PCA) included 10 variables with <40% occurrence of zero values. Variables with the highest loading for each component are in bold.

Season influenced third- and fourth-order habitat selection (Tables 3 and 4). During the active season, we primarily located gartersnakes on active or fallow pond banks or edges (60.6% of female and 41.5% of male locations) or in marshy areas of the fallow ponds (20.2% of female and 23.0% of male locations). Gartersnakes occasionally used other parts of the hatchery, such as Oak Creek or a meadow south of the ponds. On separate occasions, we located two females in semi-desert grassland habitat >100m from the ponds. On a fourth-order habitat scale, both sexes selected sloping areas close to water with a high amount of low-height cover (≤1 m in height) and vegetation, specifically forbs, and generally avoided areas with a high percentage of sedges or rushes and areas with deep water (Table 5). Females selected areas with shrubs, and males selected areas away from trees.

**Table 3:** Habitat descriptive statistics at snake points and random points across gender and season.

Variable	Female—active						Female—gestation						Male—active					
	Snake		Random		Rel.	*p*-val.	Snake		Random		Rel.	*p*-val.	Snake		Random		Rel.	*p*-val.
	Mean	SE	Mean	SE			Mean	SE	Mean	SE			Mean	SE	Mean	SE		
*Above-ground cover*																		
Canopy >1m high (%)	35.10	2.49	35.13	2.72	–	0.994	12.27	3.20	26.81	5.01	–	**0.026**	38.56	3.04	36.18	3.38	+	0.594
Low ≤1m high (%)	75.48	2.03	43.09	2.83	+	**<0.001**	45.12	4.22	33.33	5.42	+	**0.104**	80.13	2.29	48.07	3.53	+	**<0.001**
Shade (%)	76.61	2.08	52.53	2.91	+	**<0.001**	61.96	4.93	58.08	5.95	+	0.576	83.06	2.20	60.06	3.46	+	**<0.001**
Submerged veg (%)	3.32	0.97	8.84	1.77	–	**0.011**	0.01	0.01	1.55	1.49	–	0.471	1.98	1.04	4.49	1.60	–	**0.212**
*Ground cover*																		
Bare (%)	15.19	1.44	14.71	1.71	+	0.835	18.59	3.13	16.97	3.62	+	0.735	17.09	1.98	12.94	2.03	+	**0.176**
Rock (%)	7.69	1.16	14.22	1.85	–	**0.006**	12.18	2.02	10.15	2.89	+	0.609	8.07	1.61	8.92	1.73	–	0.723
Litter (%)	42.02	2.41	31.79	2.39	+	**0.003**	50.13	3.95	28.65	4.57	+	**0.002**	46.10	2.95	37.97	3.13	+	**0.051**
Woody debris (%)	0.60	0.16	0.93	0.34	–	0.390	0.14	0.06	0.40	0.27	–	0.432	0.64	0.30	1.62	0.48	–	**0.124**
Veg <1cm diam. (%)	21.27	1.80	17.20	1.98	+	**0.100**	12.08	1.73	7.11	1.93	+	**0.067**	18.86	2.17	18.50	2.39	+	0.904
Veg ≥1cm diam. (%)	3.56	0.66	3.00	0.62	+	0.481	0.37	0.26	1.66	0.57	–	**0.094**	3.96	1.10	1.69	0.49	+	**0.104**
Water (%)	16.38	2.22	24.73	2.77	–	**0.023**	1.05	0.51	32.37	5.93	–	**0.016**	18.27	3.00	22.39	3.43	–	0.353
*Vegetation*																		
None (%)	7.15	1.02	20.48	2.20	–	**<0.001**	17.38	2.59	36.68	5.48	–	**0.004**	5.61	1.26	20.46	2.87	–	**<0.001**
Grass (%)	54.95	2.37	39.03	2.52	+	**<0.001**	65.00	3.51	36.34	4.88	+	**<0.001**	52.17	3.19	43.74	3.44	+	**0.068**
Forb (%)	45.83	2.33	17.51	1.86	+	**<0.001**	37.51	3.08	13.62	3.38	+	**<0.001**	46.46	3.16	23.00	2.66	+	**<0.001**
Cattail (%)	17.95	2.40	15.58	2.35	+	0.466	1.50	1.19	11.36	4.02	–	**0.072**	21.69	3.30	10.19	2.45	+	**0.004**
Sedge/rush (%)	2.82	0.94	11.27	2.05	–	**0.001**	1.75	1.45	7.44	3.04	–	**0.147**	4.87	1.66	10.30	2.33	–	**0.078**
Shrub (%)	10.87	1.43	3.31	0.74	+	**<0.001**	2.42	0.84	4.18	2.23	–	0.455	5.36	1.02	3.95	1.25	+	0.387
Tree (%)	14.44	1.94	22.89	2.60	–	**0.008**	8.86	3.36	18.13	4.89	–	**0.136**	18.48	2.79	26.28	3.35	–	**0.064**
Aquatic (%)	5.88	1.01	9.01	1.80	–	**0.135**	1.34	0.69	1.92	1.76	–	0.761	2.72	0.90	6.00	1.94	–	**0.145**
# stems ≥1cm diam.	3.39	0.49	2.88	0.54	+	0.465	0.40	0.18	2.82	1.02	–	**0.088**	3.80	0.75	2.40	0.76	+	**0.208**
*Environmental*																		
Water depth (cm)	5.17	1.40	31.17	4.60	–	**<0.001**	0.04	0.04	39.63	9.80	–	**0.153**	5.23	1.79	26.33	5.33	–	**0.004**
Distance to water (m)	6.80	0.86	14.09	1.53	–	**<0.001**	5.70	0.67	13.29	3.03	–	**0.038**	6.22	0.91	15.82	1.68	–	**<0.001**
Aspect	145.57	23.87	55.54	57.32		**<0.001**	129.40	12.91	114.79	36.33		**<0.001**	154.12	15.17	98.04	18.14		**0.002**
Slope	12.90	0.89	5.47	0.59	+	**<0.001**	16.63	1.39	5.75	1.09	+	**<0.001**	12.62	1.15	5.75	0.74	+	**<0.001**

Direction of snake selection shown as positive or negative relative to random points. Results from univariate matched-pairs logistic regression models and variables included in multivariate models in bold.

**Table 4:** Habitat descriptive statistics at snake points and random points across gender during the inactive season.

Variable	Female—inactive						Male—inactive					
	Snake		Random		Rel.	*p*-val.	Snake		Random		Rel.	*p*-val.
	Mean	SE	Mean	SE			Mean	SE	Mean	SE		
*Above-ground cover*												
Canopy >1m high (%)	82.82	2.98	41.66	5.06	+	**<0.001**	71.77	4.34	44.04	6.28	+	**0.005**
Low ≤1m high (%)	67.40	4.04	44.15	5.29	+	**0.003**	52.30	5.14	57.19	6.26	–	0.602
Shade (%)	81.27	3.56	68.74	4.76	+	**0.055**	79.70	3.32	66.40	6.38	+	**0.077**
Submerged veg (%)	0.00	0.00	0.00	0.00	–––	–––	0.00	0.00	4.77	3.05	–––	–––
*Ground cover*												
Bare (%)	4.10	1.33	17.56	3.36	–	**0.005**	9.33	2.76	13.63	4.03	–	0.407
Rock (%)	24.57	3.53	12.47	3.08	+	**0.026**	32.64	4.37	8.45	3.09	+	**0.003**
Litter (%)	52.27	3.49	40.02	4.19	+	**0.042**	47.86	4.97	44.82	5.07	+	0.646
Woody debris (%)	2.81	0.75	1.18	0.46	+	**0.103**	2.02	0.95	0.69	0.37	+	0.289
Veg <1cm diameter (%)	14.83	2.61	17.82	2.92	–	0.453	6.86	1.25	22.19	4.16	–	**0.012**
Veg ≥1cm diameter (%)	1.13	0.60	0.62	0.34	+	0.492	1.64	0.95	1.35	0.60	+	0.797
Water (%)	0.60	0.60	10.84	3.65	–	**0.098**	0.00	0.00	10.06	4.18	–––	–––
*Vegetation*												
None (%)	2.42	0.97	20.71	4.35	–	**0.014**	2.95	1.03	12.02	3.29	–	**0.040**
Grass (%)	60.70	4.59	49.28	4.59	+	**0.101**	54.65	4.95	49.66	5.84	+	0.529
Forb (%)	23.96	4.18	17.38	3.19	+	**0.156**	36.73	5.50	17.01	4.68	+	**0.016**
Cattail (%)	0.00	0.00	4.61	2.21	–––	–––	0.00	0.00	4.65	2.73	–––	–––
Sedge/rush (%)	0.00	0.00	15.50	4.52	–––	–––	0.00	0.00	22.11	6.08	–––	–––
Shrub (%)	14.66	2.51	7.11	2.03	+	**0.022**	25.06	3.21	8.73	3.13	+	**0.003**
Tree (%)	78.68	4.01	34.77	5.44	+	**<0.001**	59.41	6.07	38.21	6.88	+	**0.049**
Aquatic (%)	0.00	0.00	0.30	0.24	–––	–––	0.00	0.00	4.20	2.85	–––	–––
# stems ≥1cm diameter	0.60	0.13	0.56	0.21	+	0.85	0.45	0.11	1.21	0.66	–	0.398
*Environmental*												
Water depth (cm)	0.05	0.05	9.41	5.01	–	0.313	0.00	0.00	10.63	6.66	–––	–––
Distance to water (m)	23.70	1.27	23.44	2.83	+	0.927	25.32	1.85	14.77	2.37	+	**0.002**
Aspect	118.79	18.80	149.41	44.05		**0.004**	155.54	10.14	141.25	15.66	–––	–––
Slope	11.75	1.31	6.43	1.08	+	**0.003**	17.07	1.88	6.79	1.30	+	**0.002**

Direction of snake selection shown as positive or negative relative to random points. Results from univariate matched-pairs logistic regression models and variables included in multivariate models in bold. Variables with a dash exhibited complete separation between snake and random locations so were omitted from multivariate analyses.

**Table 5:** Gartersnake habitat models and percent change in selection across seasons.

Variable	Weighted Coefficient	Variable Increase	Odds Ratio	% Increase/ Decrease
*Female—active*				
Low-height cover ≤1m high (%)	0.026	10%	1.299	+29.94
Distance to water (m)	-0.068	5 m	0.711	-28.85
Forb (%)	0.022	10%	1.243	+24.29
Shrub (%)	0.043	5%	1.238	+23.81
Slope (°)	0.028	5°	1.149	+14.88
Sedge/rush (%)	-0.009	10%	0.910	-8.97
Water depth (cm)	-0.003	10 cm	0.967	-3.40
*Female—gestation*				
Veg <1cm diam. ground cover (%)	0.131	5%	1.927	+92.72
Slope (°)	0.113	5°	1.760	+76.01
# of stems ≥1cm diameter	-0.226	5	0.323	-67.69
Distance to water (m)	-0.225	5 m	0.325	-67.52
Litter ground cover (%)	0.036	10%	1.432	+43.19
Canopy cover >1m high (%)	-0.021	10%	0.810	-19.02
*Male—active*				
Low-height cover ≤1m high (%)	0.039	10%	1.472	+47.24
Distance to water (m)	-0.064	5 m	0.727	-27.26
Sedge/rush (%)	-0.026	10%	0.773	-22.72
Slope (°)	0.034	5°	1.188	+18.82
Tree (%)	-0.009	10%	0.917	-8.27
Forb (%)	0.007	10%	1.077	+7.66
Water depth (cm)	-0.005	10 cm	0.950	-5.05
*Female—inactive*				
Slope (°)	0.094	5°	1.599	+59.87
Bare ground cover (%)	-0.046	10%	0.629	-37.11
Forb (%)	0.026	10%	1.301	+30.12
Canopy cover >1m high (%)	0.026	10%	1.299	+29.88
Rock ground cover (%)	0.010	10%	1.103	+10.31
*Male—inactive*				
Shrub (%)	0.065	10%	1.920	+91.95
No vegetation (%)	-0.167	10%	0.188	-81.23
Distance to water (m)	0.097	5 m	1.620	+62.05
Slope (°)	0.082	5°	1.506	+50.58
Rock ground cover (%)	0.029	10%	1.336	+33.64
Forb (%)	0.014	10%	1.154	+15.39

Results from ranked multivariate matched-pairs logistic regression models showing weighted coefficients, odds ratios, and percent change in gartersnake selection during each season. We used multiple-model inference to obtain weighted coefficients for significant variables.

During gestation, females were most often found on pond banks (78.9% of locations) or other sloping areas near the ponds (7.0%). We frequently observed them basking aboveground in mottled shade (56.1% of locations). Females selected sites close to water with a high percentage of small-diameter (<1 cm) vegetation and litter and avoided areas with a high number of large-diameter (≥1 cm) stems and a high percentage of canopy cover (Table 5).

During the inactive season, gartersnakes selected areas away from the ponds. Most gartersnakes overwintered on a rocky slope south of the ponds (49.2% of female and 73.8% of male locations) or other wooded sites (49.2% of female and 16.7% of male locations). One male overwintered on the bank of Oak Creek (9.5% of male locations). On only one occasion was a gartersnake (female) located in an area with water in the plot (1.5% of female inactive locations; <1% of all inactive locations). On a fourth-order habitat scale, both sexes selected rocky slopes with a high percentage of forbs (Table 5). Females selected areas with a high percentage of canopy cover (>1 m in height) and avoided areas with a high amount of bare soil ground cover. Males selected areas farther from water with a high amount of vegetation, especially shrubs. Most transmittered gartersnakes went through a transition period just prior to and after the inactive season, during which they moved between their overwintering areas and the ponds multiple times before settling into their core overwintering areas.

### Activity ranges

Both sexes occupied larger areas during the active season than during other seasons, and males generally had larger activity ranges than females. Females infrequently moved >10m in a week during the gestation season, and we often found them in the same location as the previous week. Most snakes went through a transition period just prior to and after the inactive season (Table 6), during which they moved between their overwintering areas and the ponds multiple times before settling into their core overwintering areas. The length of this transition period varied by individual snake but generally occurred in September–November and February–April. After settling into their core overwintering areas, females rarely moved >10m during a week, but four of six males included in the activity-range analyses regularly moved >10m during a week in the inactive season.

**Table 6:** Area of gartersnake activity range.

Season	Size	Female						Male					
		n	Mean	Median	SE	Range		n	Mean	Median	SE	Range	
						Min	Max					Min	Max
Active	m^2^	9	3319.47	2438.00	766.29	297.00	10314.00	4	7638.79	4080.50	3508.21	330.00	28104.50
	ha		0.33	0.24	0.08	0.03	1.03		0.76	0.41	0.35	0.03	2.81
Gestation	m^2^	7	57.64	35.50	26.26	3.00	207.00	–––	–––	–––	–––	–––	–––
	ha		0.01	0.00	0.00	0.00	0.02						
Inactive + Transition	m^2^	10	969.35	38.00	531.08	11.50	4257.00	6	1401.17	72.75	1279.64	1.00	7790.50
	ha		0.10	0.00	0.05	0.00	0.43		0.14	0.01	0.13	0.00	0.78
Core inactive	m^2^	10	10.50	6.50	2.37	3.50	23.00	6	103.28	36.75	70.22	1.00	447.00
	ha		0.00	0.00	0.00	0.00	0.00		0.01	0.00	0.01	0.00	0.04

Descriptive statistics for activity ranges (m^2^ on top line, ha on second) calculated by season using 95% minimum convex polygons. Inactive season was further broken down into inactive + transition period, which includes movements at the beginning and end of the inactive season. The core inactive season represented when snakes settled into a small overwinter area. Number of individuals (n) per season includes individuals for which activity range size plotted against number of locations reached an asymptote (active: minimum of 6–16 locations; gestation: 3–7 locations; inactive: 3–14 locations). During the core inactive season, some males continued to move, and activity range size plotted against number of locations did not reach an asymptote; all locations were used in these cases.

### Body size, temperature, and condition

**Fig 5:**
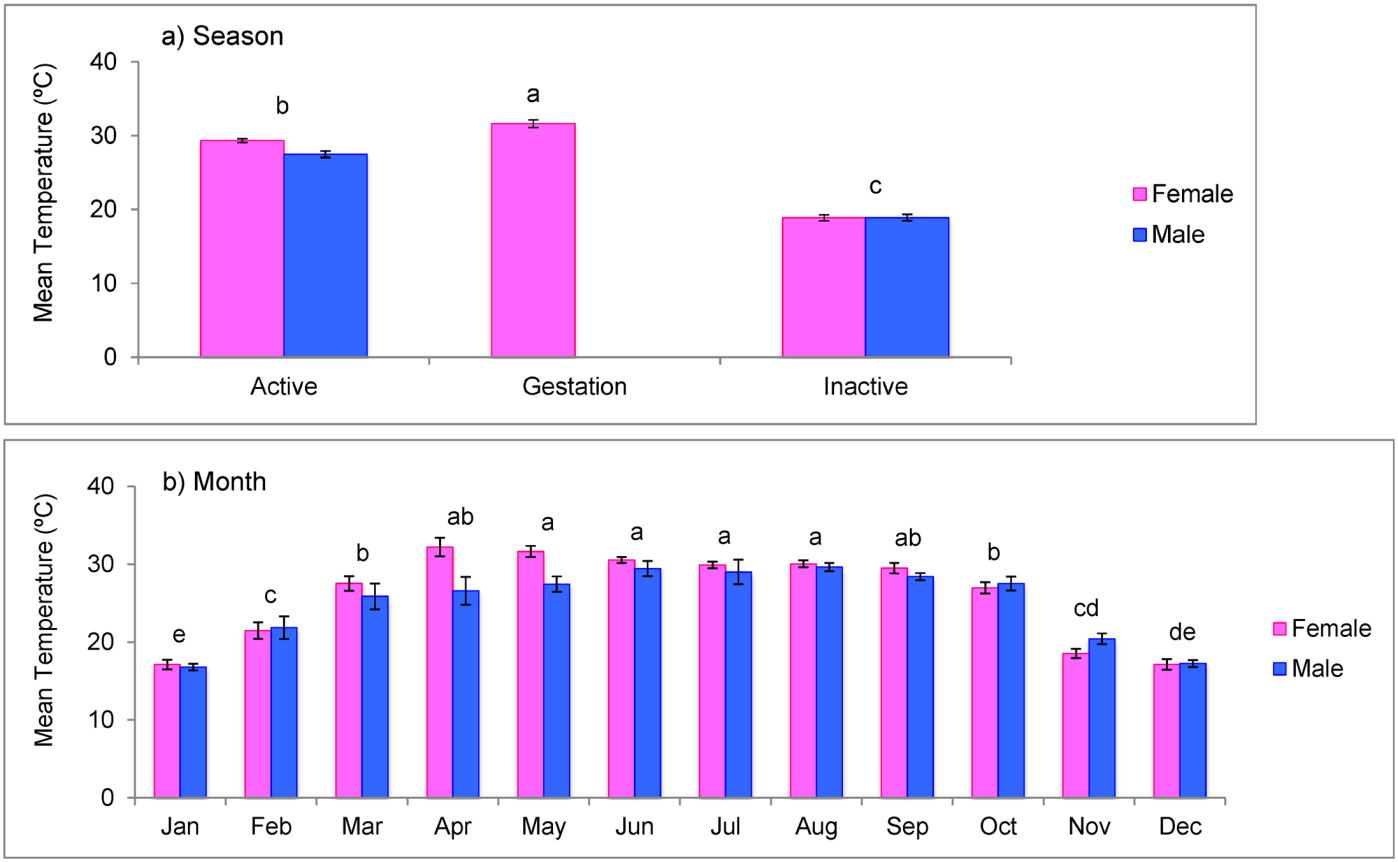
Mean gartersnake body temperatures calculated from temperature-sensing transmitters by a) season and b) month. Bars show standard error and letters represent significant differences between seasons/months from mixed-effects ANOVAs. Sex was not a significant factor.

Females were larger than males. Female mass ($\bar{\text{x}}=222.9\pm 20.2\;\text{g}$) was greater than males ($\bar{\text{x}}=92.9\pm 4.9\;\text{g}$; F = 6.255, *p*<0.001). Snake body temperature, as calculated from transmitter pulse rate, varied by season (season: F = 418.750, df = 2, 685, *p*<0.001; sex: F = 2.410, df = 1, 31, *p* = 0.131) and by month (month: F = 96.048, df = 11, 671, *p*<0.001; sex: F = 0.445, df = 1, 32, *p* = 0.510) but not by sex. Females were warmest during the gestation period ($\bar{\text{x}}=31.6{}^{\circ}\text{C}$); both sexes were cooler during the inactive season ($\text{female}\;\text{and}\;\text{male}\;\bar{\text{x}}=18.9{}^{\circ}\text{C}$) compared to the active season ($\text{female}\;\bar{\text{x}}=29.3{}^{\circ}\text{C}$, $\text{male}\;\bar{\text{x}}=27.5{}^{\circ}\text{C}$; Fig 5a). On a monthly basis, gartersnakes were warmest from May–August and coolest from December–January (Fig 5b).

Ten transmittered gartersnakes (23.8%) exhibited short-term signs of illness, including infection at the transmitter site, a herniated transmitter, or poor body condition. To determine if these illnesses affected our results, we compared means and standard errors of habitat variables from locations of sick animals with the remaining data and found that the overall pattern of habitat selection did not vary. Therefore, we included all locations in habitat analyses.

## Discussion

Our approach of using radio telemetry to monitor gartersnakes across seasons provides an assessment of habitat selection for a threatened subspecies occupying a highly-managed environment. In this study, gartersnakes displayed distinct habitat selection during three seasons: active (March–October), gestation (April–May), and inactive (November–February). We found that gartersnakes displayed more precise habitat selection during winter and during the period for female gestation. Gender did not substantially contribute to differences in habitat selection. Notably, this hatchery can be used to identify spatial and temporal patterns important for conservation of this subspecies across seasons.

### Active season

During the active season, snakes must select areas that provide resources for growth and survival [23]. In our study, northern Mexican gartersnakes primarily selected wetland edges during the active season, including active and fallow pond banks and edges. These areas provided access to foraging opportunities and basking sites while also providing cover and abundant rodent burrows for thermoregulation and protection from predators. Both females and males selected sloping sites close to water with dense vegetation and low-height cover. Females were more often found near shrubs, which might provide important cover, and males were rarely found near trees. These results appear to be consistent with preliminary findings from more-natural habitats in central Arizona. Our colleagues [39] found that gartersnakes selected sloping areas at aquatic edges with dense emergent vegetation.

In our study, gartersnakes used marshy habitats in the fallow ponds to a lesser extent than pond edges. These marshy habitats offered abundant cover and access to prey, including amphibians. Studies of other species of gartersnakes have documented use of marshy habitat for cover and prey [13,55]. Our colleagues [40] suggested fallow ponds at the hatchery might be most important following amphibian breeding in spring and during the monsoon (July to early September [74]). However, in our study, gartersnakes consistently used fallow ponds throughout the active season, perhaps selecting more for cover characteristics than for foraging opportunities.

Selection of dense cover has been documented for several species of gartersnakes [6,14,55], watersnakes [53], vipers [1], and pythons [75]. Low-height cover might be especially important at the hatchery because it supports high numbers of predators, including raptors, herons, and bullfrogs [40]. Cover along pond banks was not static during our study because hatchery personnel occasionally trimmed or removed vegetation along banks of fish-rearing ponds. After vegetation removal, gartersnakes responded to this habitat alteration by relocating to more vegetated banks or to unaffected areas of the hatchery close to water.

Our data were consistent with studies that found proximity to water is important for other species of snakes [1,6,13,45]. Proximity to water provides gartersnakes with foraging opportunities and an escape from terrestrial predators. We observed both of these behaviors at the hatchery, and gartersnakes would occasionally flee from observers into ponds. Although both sexes generally used pond shallows, gartersnakes occasionally used deeper sections of ponds for foraging and possibly for thermoregulation. Some studies have documented snakes using water to regulate body temperatures [45,76,77].

### Gestation season

During gestation, females exhibited similar third-order habitat selection as during the active season but selected different fourth-order habitat features. The most notable difference was cover. Females avoided canopy cover, and cover ≤1 m in height was not important. We often observed females basking or located them underground in sites exposed to sun. Elevated body temperatures calculated from transmitter pulse rates indicated that gestating females selected areas for thermal qualities. Pregnant females thermoregulate more precisely and typically at higher temperatures than non-pregnant snakes [7,78] and often select sites with optimal sun exposure and heat [79]. For example, we commonly located two females under black pond liners where temperatures were generally warmer than the surrounding area. In addition to thermoregulation needs, viviparous snakes also experience reduced locomotor ability due to developing embryos [6,80], which presents a trade-off between thermoregulation and predator avoidance. At the hatchery, females selected sites that appeared to satisfy both needs–close to open areas for basking but near dense vegetation or rodent burrows for escape from predators.

During gestation, females continued to select sloping areas close to water, primarily pond banks. Although females of many species of snakes cease foraging during the latter part of gestation [8,81], pregnant females often choose sites close to water. Partially because of this late-term feeding avoidance, post-parturient snakes often appear emaciated [6,81,82] and might select areas close to foraging opportunities for after they give birth [7]. Females also might require increased water intake during gestation [83]. Another possibility is that selection of sites near water could be important for neonates. For example, perhaps females of aquatic species give birth near water to facilitate shedding of neonatal skin with minimal water loss or to place neonates in close proximity to habitats with suitable prey. Postpartum females in our study resumed activities in hatchery ponds.

### Inactive season

During the inactive season, gartersnakes selected rocky slopes or woodlands more distant from ponds. In comparison, our colleagues [39,41] found that northern Mexican gartersnakes in more-natural areas used a variety of overwinter sites, including upland habitats, meadows, and aquatic edges. These studies and ours indicate that northern Mexican gartersnakes commonly overwinter in upland habitats, although riparian floodplains and water edges are also used to a lesser extent. Use of terrestrial, upland habitats has been documented for a variety of semi-aquatic herpetofauna [11,45,84], perhaps due to thermoregulatory benefits or to avoid potential flooding events during the winter. However, importance of upland sites is often overlooked for semi-aquatic species [84]. Habitat modifications and soil compaction that occur in these upland areas could have negative effects on overwintering success of gartersnakes. Gartersnakes in our study also exhibited more precise selection of habitat parameters during the inactive season compared to active season. Others [5] suggest that precise selection of overwintering sites can be more important than site selection during the active season because overwinter sites that do not provide adequate resources might result in reduced fitness or mortality. Because of this specific selection of habitat, individuals might repeatedly use the same overwintering sites, which has occasionally been observed in other studies with this subspecies [39,40].

During the inactive season, females and males selected areas in close proximity to each other with some differences in specific habitat features, possibly due to variation in thermal qualities [85] and subterranean characteristics [86,87]. Females selected areas with a high percentage of canopy cover, whereas this variable was not as important for males. Perhaps due to their smaller body size, male gartersnakes might have selected sites with more sun exposure and warmth in order to maintain body temperature [86,88]. Body temperatures were similar between sexes during the inactive season. Because body size and temperature are closely linked, larger individuals maintain heat longer [89,90] and females might have selected sites protected from daily temperature changes. The smaller body size of males might also have enabled them to inhabit a wider variety of subterranean sites, whereas females might have made use of burrow systems provided by tree roots or by rodents associated with those roots [87,91,92].

### Animal health

Studies of rare species or species of conservation concern often involve conducting research using wild-caught animals. Researchers can be faced with ethical dilemmas when balancing the potential harm of research with the benefits gained from understanding how to conserve species and their habitats [93]. Animal conservation research is designed to understand aspects of an animal’s biology or ecology with the hope of identifying how to safeguard these species. Some conservation efforts have made great strides (such as understanding and mitigating white-nose syndrome in bats [93]) by studying animals to understand their ecology and ecosystems. Researchers must consider effects of their work on study animals and populations [94,95].

Compared to other northern Mexican gartersnake research [39–41], we observed a high occurrence of illness, mortality, and premature failure of telemetry units in transmittered snakes, even though implant and tracking methods were similar. However, limited information is available on the effect of transmitters on snake morbidity and survival. Telemetry could have negatively affected survival in black ratsnakes (*Elaphe obsoleta*) [96]. Others [97] reported increased infection and inflammatory response in eastern massasauga rattlesnakes (*Sistrurus catenatus catenatus*) implanted with transmitters. Methods such as telemetry represent an important trade-off between possible negative effects to individual animals or populations and understanding species ecology and management needs [98].

The use of telemetry can provide researchers with data unattainable using other methods, such as visual encounter-type surveys. Telemetry has been found to be an effective way to track some species of snakes without impacting their movement patterns [99]. It is important to determine which monitoring techniques are best to achieve a specific conservation goal [98], such as identifying critical habitat for species recovery. Regardless of technique, researchers should consider how robust a population is and what proportion of individuals will be included in the study. Researchers should monitor indicators of stress during studies [100]. We also suggest monitoring for external signs of illness and infection by assessing body condition periodically during research. Animals that exhibit abnormal levels of stress, signs of illness, or poor body condition should, if appropriate, receive medical attention and have monitoring devices removed. Every project is unique, and it is incumbent upon researchers to minimize negative outcomes for populations and individuals to the best of their ability, especially when working with imperiled species.

## Conclusions and implications

Incorporating habitat needs of northern Mexican gartersnakes into development and resource management plans is an essential component of ensuring that populations of this subspecies are maintained or restored [2,4]. As the human population continues to grow, demand for land and water will also increase [26], causing profound effects on riparian habitats and the species that depend on these areas [25,26,42]. Management decisions occurring within the range of northern Mexican gartersnakes must take into account the full range of third- and fourth-order habitat parameters required for this subspecies, which includes needs during different seasons and physiological periods. Conservation of this subspecies requires a landscape-level approach that incorporates protection of wetlands, including abundant wetland edge habitat, and connected terrestrial upland both adjacent to and more distant from these wetlands [39,40,42,84]. Connectivity of these areas is vital. Just prior to and after the inactive season (October–November and February–March), many gartersnakes moved between their overwinter sites and the ponds several times before settling into their overwinter habitat. Managers should be aware of such movements and should stage activities (such as moving heavy equipment or vehicles on roads around ponds and overwintering sites) to avoid disturbing gartersnakes that may be active during these times.

Managers should maintain structural diversity of the habitat, including varying degrees of cover density and height. For example, sites close to water with dense vegetative cover should be provided for thermoregulation and predator avoidance during the active season. Adjacent open or less-densely vegetated areas for basking are beneficial during the active and gestation seasons. Rocky slopes that offer a mix of open and closed tree or shrub canopy are necessary for the inactive season. Habitat modifications that occur in these areas, including when gartersnakes are not currently using them, could have profound effects on individuals or the population. Large-scale vegetation removal or soil compaction activities should be avoided. Changes that occur in overwintering habitat may be especially detrimental, considering the precise fourth-order habitat selection we observed. Further research to determine if this subspecies exhibits overwinter site fidelity over multiple years would benefit management decisions.

Bubbling Ponds Hatchery is one of five known viable populations of northern Mexican gartersnakes in the United States [32]. Therefore, management of this site for northern Mexican gartersnake conservation is important, and suitable resources for the population should be maintained. As habitat at the hatchery changes resulting from human activities and ecological succession (such as invasion of trees in fallow ponds, altering the marsh-like characteristics of this habitat), understanding how this population responds to changes in habitat can help inform management decisions across this subspecies’ range. The hatchery study site could serve as a model for restoration or management of similar areas, especially where human-constructed ponds provide prey and adequate cover along pond edges. However, some habitat features provided by the hatchery area may not translate into more natural riparian and wetland areas with lentic or intermittent flows. Further research in more natural systems can help determine appropriate management techniques. Interestingly, this subspecies appears to be thriving in a highly-modified and heavily-used area that also supports abundant predators. This population provides an example of where, even as development and human activities continue, the gartersnake is able to persist. We recommend careful land use planning to maintain areas where appropriate resources provide habitat for the northern Mexican gartersnake.

## Supporting information

S1 Fig Northern Mexican gartersnake. A female northern Mexican gartersnake (*Thamnophis eques megalops*) during the gestation season.
(JPG)

S1 Table Transmittered gartersnakes included in the study. Mass was averaged for snakes captured more than once. Snakes received internal (I), external (E), or both (I/E) types of transmitters. Number of locations includes all locations for that gartersnake. Months tracked not continuous for all snakes due to shed transmitters. Mean (±SE) mass for females and males are shown in the bottom rows. A one-way t-test was used to test if female mass was greater than male mass.
(DOCX)

S1 Dataset Habitat measurements collected at snake and random locations, body temperature data. (XLSX)
