# Supplementary figures and images for: Influence of seasonality and gestation on habitat selection by northern Mexican gartersnakes (Thamnophis eques megalops)
Source: PLoS One. 2018 Jan 30;13(1):e0191829. doi: 10.1371/journal.pone.0191829 (PMC5790243; doi:10.1371/journal.pone.0191829)

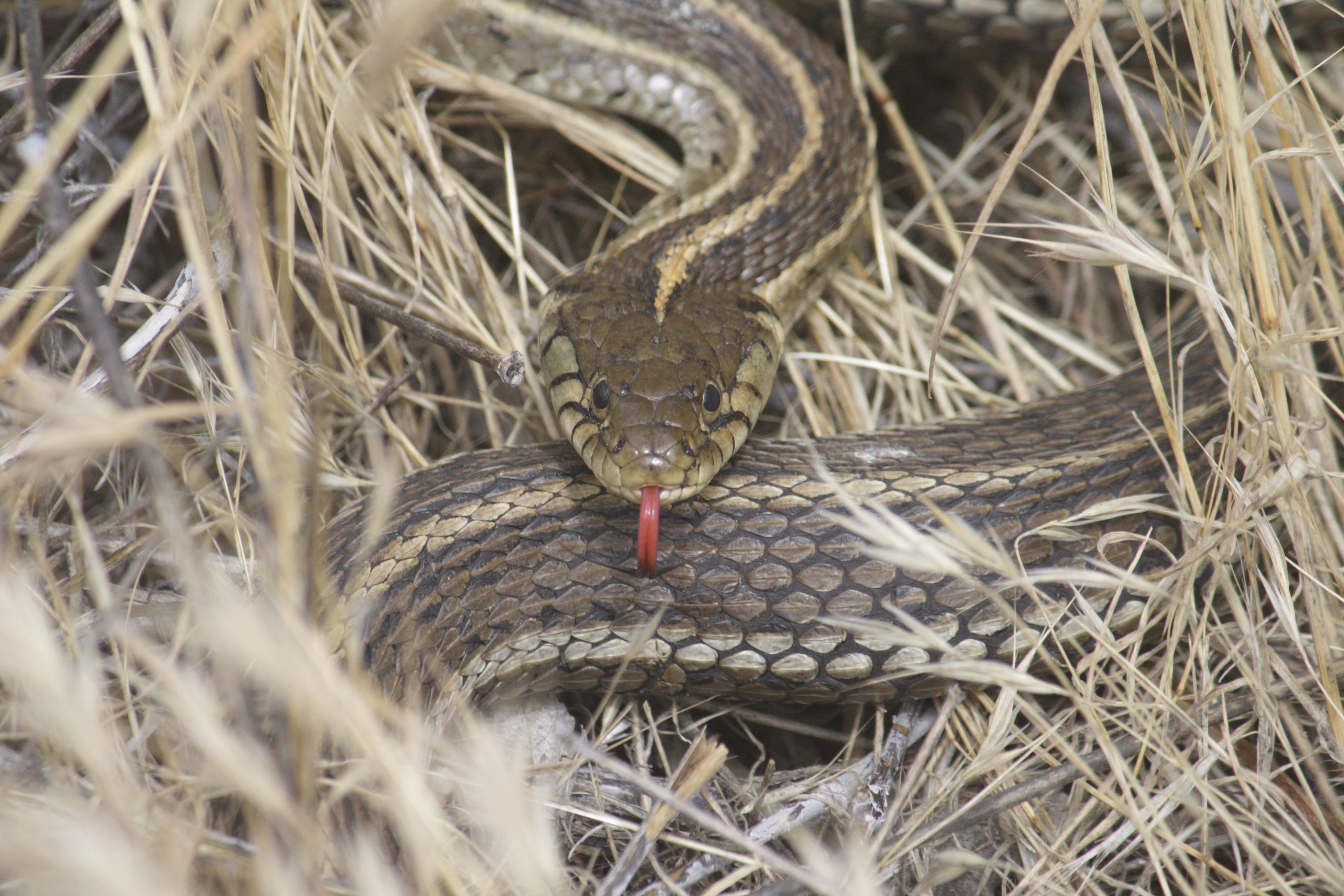

Supplement: S1 Fig — A female northern Mexican gartersnake (Thamnophis eques megalops) during the gestation season. (JPG) [file pone.0191829.s001.JPG]
