# Supplementary material for: Influence of seasonality and gestation on habitat selection by northern Mexican gartersnakes (Thamnophis eques megalops)
Source: PLoS One. 2018 Jan 30;13(1):e0191829. doi: 10.1371/journal.pone.0191829 (PMC5790243; doi:10.1371/journal.pone.0191829)
Supplement: S1 Table — Mass was averaged for snakes captured more than once. Snakes received internal (I), external (E), or both (I/E) types of transmitters. Number of locations includes all locations for that gartersnake. Months tracked not continuous for all snakes due to shed transmitters. Mean (±SE) mass for females and males are shown in the bottom rows. A one-way t-test was used to test if female mass was greater than male mass. (DOCX) [file pone.0191829.s002.docx]

| **Snake** | **Sex** | **Mass (g)** | **Transmitter Type** | **# of  Locations** | **Months Tracked** | **Fate** |
| --- | --- | --- | --- | --- | --- | --- |
| 19 | F | 425.0 | I | 5 | 1.8 | Mortality (likely predation) |
| 20 | M | 100.5 | E | 4 | 0.3 | Shed transmitter |
| 21 | F | 212.0 | I/E | 15 | 2.9 | Mortality (cause unknown) |
| 22 | M | 117.0 | I/E | 17 | 7.0 | Expelled transmitter |
| 23 | F | 324.5 | I/E | 30 | 10.5 | Removed transmitter |
| 24 | M | 89.0 | E | 4 | 0.4 | Shed transmitter |
| 25 | M | 91.3 | E | 7 | 1.3 | Shed transmitter |
| 26 | M | 108.7 | I | 22 | 4.9 | Signal lost |
| 27 | F | 216.6 | I | 36 | 13.3 | Removed transmitter |
| 28 | F | 191.0 | I | 4 | 0.5 | Mortality (likely predation) |
| 29 | F | 293.8 | I/E | 29 | 11.7 | Removed transmitter |
| 30 | F | 139.0 | E | 2 | 0.2 | Shed transmitter |
| 31 | F | 82.0 | E | 1 | 0.1 | Shed transmitter |
| 32 | M | 108.5 | E | 6 | 0.9 | Shed transmitter |
| 33 | F | 455.0 | I/E | 30 | 9.6 | Removed transmitter |
| 34 | F | 121.0 | E | 3 | 0.4 | Shed transmitter |
| 35 | F | 324.8 | I/E | 27 | 10.8 | Mortality (cause unknown) |
| 36 | M | 69.0 | E | 4 | 0.5 | Shed transmitter |
| 37 | M | 103.0 | I/E | 22 | 8.8 | Signal lost |
| 38 | F | 325.0 | I | 2 | 0.9 | Mortality (cause unknown) |
| 39 | F | 191.0 | I | 27 | 11.2 | Removed transmitter |
| 40 | F | 291.0 | I | 18 | 7.0 | Removed transmitter |
| 41 | M | 114.8 | I | 23 | 9.3 | Signal lost |
| 42 | F | 269.5 | I | 30 | 10.8 | Signal lost |
| 43 | M | 103.7 | I | 26 | 10.1 | Signal lost |
| 44 | M | 71.5 | E | 1 | 0.2 | Shed transmitter |
| 45 | M | 52.3 | E | 2 | 0.4 | Shed transmitter |
| 46 | M | 64.0 | E | 4 | 0.7 | Shed transmitter |
| 47 | F | 188.0 | I | 22 | 9.4 | Removed transmitter |
| 48 | M | 115.0 | I | 5 | 1.2 | Signal lost |
| 49 | F | 238.0 | I | 20 | 8.2 | Unknown (found transmitter) |
| 50 | M | 101.5 | I/E | 16 | 7.5 | Removed transmitter |
| 51 | M | 69.2 | E | 13 | 5.2 | Mortality (likely predation) |
| 52 | F | 98.0 | E | 4 | 1.2 | Shed transmitter |
| 53 | F | 254.0 | I | 2 | 1.0 | Signal lost |
| 54 | F | 132.0 | E | 3 | 1.8 | Shed transmitter |
| 55 | F | 275.0 | E | 4 | 1.1 | Shed transmitter |
| 56 | F | 89.5 | E | 2 | 0.7 | Shed transmitter |
| 57 | F | 134.0 | E | 4 | 0.7 | Shed transmitter |
| 58 | F | 115.0 | E | 1 | 0.2 | Shed transmitter |
| 59 | F | 188.0 | E | 4 | 0.7 | Shed transmitter |
| 60 | M | 100.5 | E | 1 | 0.2 | Shed transmitter |
| Mean (female) | | 222.9 (20.2) | |  |  |  |
| Mean (male) | | 92.9 (4.9) | |  |  |  |
| One-tailed t-test | | **t=6.255, *p*<0.001** | |  |  |  |
